# Separation and characterization of nitrated variants of the major birch pollen allergen by CZE-ESI-μTOF MS

**DOI:** 10.1002/elps.201300151

**Published:** 2013-08-21

**Authors:** Sergey Gusenkov, Chloé Ackaert, Hanno Stutz

**Affiliations:** 1 Division of Chemistry and Bioanalytics, Department of Molecular Biology, University of SalzburgSalzburg, Austria; 2 Division of Allergy and Immunology, Department of Molecular Biology, University of SalzburgSalzburg, Austria

**Keywords:** Allergen, CZE, ESI-TOF MS, Nitration, Peroxynitrite

## Abstract

A CZE-ESI-TOF MS method has been optimized for the separation and identification of nitrated variants of the major birch pollen allergen from *Betula verrucosa*, isoform 1a (Bet v 1a). In-house nitration of recombinant Bet v 1a was done by peroxynitrite. As a BGE, 10 mmol/L ammonium bicarbonate with pH 7.50 provided best resolution. Nebulizer gas pressure and sheath liquid flow rate of 0.4 bar and 6 μL/min, respectively, maintained CZE selectivity and constituted stable electrospray conditions. A sheath liquid composition of 75% v/v methanol with 0.1% v/v formic acid in ultrapure water resulted in highest signal intensities. Alternatively, methanol could be replaced by 50% v/v isopropanol. Two modified allergen products derived from reaction mixtures that contained different amounts of the nitration reagent were compared by the elaborated CZE-ESI-TOF MS method. Up to twelve different Bet v 1a variants with one- to sixfold nitration could be distinguished. Several allergen fractions of equivalent nitration grade were resolved. Their different migration times indicate site-specific nitration with concomitant differences in p*I* and maybe also in hydrodynamic radius. The method allows for a characterization of in-house nitrated allergen samples that are intended for testing the postulated enhanced allergenicity of nitrated Bet v 1a variants.

## 1 Introduction

Allergies represent misguided immunoglobulin E-mediated immune responses against physiologically harmless proteins [1]. About 20–30% of the European population suffer from respiratory allergies, elicited by airborne allergens primarily released from tree and grass pollen [2]. The pronounced increase of allergic disorders indicates a relation between increasing allergy prevalence and environmental air pollutants, namely NO_2_ and O_3_ [3–5]. Besides, nitration can also occur in vivo triggered by peroxynitrite (PN) during tissue inflammation [6]. Protein nitration is also indicative for nitrosative stress [7], occurs in defense strategies against alien proteins and pathogens [5, 8] and might compete with phosphorylation [8]. In addition, nitration can induce shifts in protein conformation [8, 9], entailing changes or even loss in the protein function or an increase in the susceptibility to proteases [8, 10, 11]. Increased nitration by PN has also been discussed for neurodegenerative diseases, e.g. Alzheimer's disease [12].

Up to 54% of allergic patients are sensitized against birch pollen allergens stemming from birch tree *Betula verrucosa* with the major isoform 1a, i.e. Bet v 1a, representing one of the most prominent aero-allergens [13]. Nitration of Bet v 1a was shown to enhance its allergenicity in comparison to the nonmodified wild-type [14]. Recent results have indicated a higher diversity and abundance of peptides that are derived from nitrated Bet v 1a (nBet v 1a) and are exposed by antigen presenting cells after processing nBet v 1a [15]. In vitro nitration of Bet v 1a has been employed either by tetranitromethane or PN [6, 16]. PN primarily addresses cysteine, methionine, or tyrosine (Tyr) [17]. Tyr is modified by degradation products of PN. In the presence of CO_2_, the decay of PN occurs in a fast reaction via an instable intermediate, i.e. peroxycarbonate, according to the following equation [6, 18]:


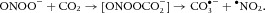


The solvent-caged radicals CO_3_^•−^ and •NO_2_ are partly released and nitrate Tyr in two steps first forming a Tyr radical (Tyr•) that is then nitrated to 3-nitro-Tyr by •NO_2_ [19]. Nitration propensity of Tyr is protein and site specific and related to the surface exposure and the molecular environment [17, 19]. Nitration entails a reduction in the pK_a_ from 10.0 to 7.5 in free Tyr [6] inducing shifts in p*I* and possibly conformational changes [11]. In total, Bet v 1a contains seven Tyr residues (http://www.uniprot.org/uniprot/P15494). Thus, multi-site nitration of the target protein will create complex profiles of numerous closely related variants and might also modify impurities or degradation products contained in the initial source material. Therefore, a separation system highly selective for changes in p*I* and conformation is required for the characterization and quality control of nitrated allergen products.

Commonly, HPLC with UV- or MS detection has been applied to address nitration of proteins either in intact form or on peptide level [20–23]. Remarkably, only one CZE-UV separation has been reported in this context to date [10], although CZE seems predestined for this task since it can equally address minute changes in the protein net charge and the conformation [24–27]. The applicability of various CE-UV modes, such as CZE, CIEF, and affinity CIEF, for the characterization of modified recombinant allergens has been described previously [28–32]. In comparison with UV, MS detection exhibits a higher sensitivity and allows for a further confirmation of identity by mass information of intact allergen variants. Within the arsenal of analytical tools, CZE-ESI-MS has demonstrated its remarkable potential for protein characterization and profiling over the last decade [33–39]. TOF MS is frequently employed, particularly in proteome analysis, due to its high sensitivity, high scan rate, excellent mass accuracy, and resolution of intact proteins [33, 35, 40, 41]. The hyphenation of CZE to MS requires volatile BGEs that restrict the spectrum of applicable BGEs and modifiers [42–44]. Among CE-MS coupling strategies, “sheath liquid interface” is still considered a highly robust and stable sprayer in comparison to “liquid junction” and “sheath less” modes [42, 45, 46]. The recently developed porous sprayer provides numerous advantages [47, 48], but lacks universal compatibility with all commercial instruments. One of the disadvantages associated with “sheath liquid” (SL) sprayer is the unavoidable addition of ionic and neutral species that may together with the dilution effect impair sensitivity [44]. Thus, a comprehensive optimization is recommended to reduce the contribution of aforementioned effects. Several interrelated parameters, e.g. nebulizer gas (NG) pressure, SL composition and flow, and BGE concentration, are crucial for gaining an effectual electrospray providing stability and sensitivity [49, 50].

Nitrated allergen products are commercially not available to date, to say nothing of their standardization. Thus, this work aims to develop and optimize a CZE-ESI-TOF MS method capable to handle heterogeneous mixtures of nBet v 1a variants sorted over a narrow p*I* range. This targets profiling, identification of the nitration grade of intact proteins, at best indirect distinction of nitration-sites based on migration times (*t*_m_), identification of remaining nonmodified Bet v 1a, and efficacy control of the nitration reaction. Identification of minor variants and possible nitrated impurities requires a highly selective separation in combination with high mass resolution. CZE-ESI-TOF MS characterization of in-house produced nBet v 1a is intended as a control for reaction optimization and final characterization to ensure consistent product composition. This is a prerequisite for a correct interpretation of biological effects of nBet v 1a derived by immunologists to gain a better insight into allergic pathways underlying nitrated variants.

## 2 Materials and methods

### 2.1 Chemicals

Ammonium bicarbonate (in eluent quality for LC-MS), formic acid (FA, 98–100%), methanol, isopropanol (IP) (both for LC-MS) and diethylenetriaminepentaacetic acid (99%) were purchased from Sigma-Aldrich (Steinheim, Germany). A 25% v/v ammonia solution, 85% w/v ortho-phosphoric acid (both in p.A. quality), and a 1.0 mol/L NaOH solution were purchased from Merck (Darmstadt, Germany). A 1.0 mol/L HCl solution was obtained from AppliChem (Darmstadt, Germany). Na_2_HPO_4_ (in p.A. quality) was from Carl Roth (Karlsruhe, Germany). Sodium peroxynitrite solution in 100–150 mmol/L NaOH was purchased from Cedarlane (Burlington, Ontario, Canada). Ultrapure water was supplied in a quality higher than 18.2 MΩ.cm by a Milli-Q Plus 185 system (Millipore S.A., Molsheim, France). ESI-L Low Concentration Tuning Mix was from Agilent Technologies (Santa Clara, CA, USA) and used as received for calibration of micrOTOF MS. Nitrogen in 5.0 quality was obtained from SIAD (St. Pantaleon, Austria) and applied as dry gas (DG) and NG in MS.

### 2.2 Allergens

Recombinant Bet v 1a was produced in-house and kindly provided. Production and purification of recombinant Bet v 1a was described elsewhere [51]. Bet v 1a in-house standard was reconstituted in 10 mmol/L phosphate buffer to provide 4.0 mg/mL and then split in 20 μL aliquots that were stored at –20°C until their analysis. Concentration was derived by amino acid analysis. To avoid repetitive freezing and thawing, aliquots thawed for analysis were stored at +4°C until their consumption within one week.

### 2.3 Nitration of Bet v 1a with PN

For nitration of Bet v 1a, the allergen was reconstituted in 50 mmol/L ammonium bicarbonate (pH 7.8), containing diethylenetriaminepentaacetic acid at final 0.1 mmol/L, to provide a final allergen concentration of 1.0 mg/mL. Nitration was performed with sodium peroxynitrite, according to the manufacturer's guidelines: therefore, PN was thawed slowly and used immediately. Nitration was accomplished by reagent addition to obtain 1:1 or 5:1 molar ratios between PN and Tyr residues in Bet v 1a. The reaction mixture with PN was stirred for 1 h at room temperature and afterwards centrifuged over Amicon centrifugal filter units with a molecular cut-off of 10000 (Merck Millipore, Cork, Ireland) to separate Bet v 1a from PN. The allergen was washed three times by adding fresh 10 mmol/L phosphate buffer, adjusted to pH 7.4 with ortho-phosphoric acid and finally reconstituted in 500 μL 10 mmol/L phosphate buffer.

### 2.4 Preparation of BGE

An appropriate amount of ammonium bicarbonate was weighed in a 100-mL volumetric flask and dissolved in ultrapure water. For the finally optimized 10 mmol/L BGE, 79.10 ± 0.20 mg of ammonium bicarbonate were weighed. The required pH was adjusted immediately prior to use in CZE-ESI-TOF MS either by ammonia or FA. The pH was measured by means of a WTW Microprocessor pH 3000 pH meter from WTW (Weilheim, Germany) calibrated with Hamilton Duracal buffers with pH 7.00 and 10.01 (Hamilton Bonaduz, Bonaduz, Switzerland).

### 2.5 Preparation of SL

Preparation of the SL was accomplished by mixing the selected organic solvent with ultrapure water in an appropriate ratio in a 100 mL graduated cylinder. The mixture was then transferred to a glass Duran bottle with subsequent addition of FA according to the selected SL composition (see text).

### 2.6 Instruments

#### 2.6.1 Capillary electrophoresis

CZE was carried out with a P/ACE™ System MDQ CE of Beckman Coulter (Brea, CA, USA) operating under 32 Karat software version 7.0. Separation was performed in bare fused silica capillaries of 50 μm id and 365 μm od with a total length (L_T_) of 100.5 cm (Polymicro Technologies; Phoenix, AZ, USA). The capillary bypassed the UV detector and detection was only by TOF MS. Prior to first use, new capillaries were conditioned as described elsewhere [52]. Briefly, capillaries were rinsed by a sequence of 1.0 mol/L NaOH (10 min), ultrapure water (15 min), 0.1 mol/L HCl (10 min), and BGE (20 min), all with 1500 mbar. Finally, the capillary was conditioned for 10 min with +15.0 kV when filled with BGE. Every three runs, the capillary was rinsed with 0.10 mol/L NaOH solution (2 min), ultrapure water (2 min), 0.10 mol/L HCl (2 min), ultrapure water (2 min), and finally with BGE (2 min), all with 1500 mbar [52] with the ESI probe decoupled. Subsequent to the final BGE rinsing, +15.0 kV were applied for 1 min. Hydrodynamic sample injection was done at 35 mbar for 10 s. CZE separations were performed at +25.0 kV with the cathode situated at the capillary outlet selecting a ramp time of 1.0 min. Temperature of capillary cartridge was set to 25.0°C. Capillaries were stored in BGE when not in use and overnight.

#### 2.6.2 CZE-ESI-μTOF MS

The CE system was hyphenated to a micrOTOF MS of Bruker Daltonics (Bremen, Germany) by means of a coaxial CE-ESI-MS sprayer of Agilent Technologies. SL was delivered by a MicroProEldex syringe pump (Napa, CA, USA) at various appropriate flow rates (see text). Tuning of the ion optics to optimize MS signal intensity was done by introducing the allergen target product, i.e. nitrated Bet v 1a, at 0.4 mg/mL with 100 mbar via the separation capillary with simultaneous application of SL. Manual calibration was performed with undiluted ESI-L low concentration tuning mix of Agilent Technologies by a predefined reference mass list. MS detection was in positive polarity mode with –4.50 kV applied to the MS transfer capillary whereas the sprayer was set to ground. Endplate offset was –500 V. Transfer and PrePuls of Lense 1 were selected with 80 μs and 20 μs, respectively. A flow rate of 4 L/min and a temperature of 190°C were selected for the DG. Data treatment was done with Data Analysis software, Version 4.0 SP 1, from Bruker Daltonics. Deconvolution was done by the Maximum Entropy option with data point spacing of 0.1 and a mass resolution of 10000.

## 3 Results and discussion

Some detrimental phenomena, i.e. suction and dilution effects, are associated with the design of the frequently used coaxial SL interface [49, 50]. Thus, the development of a CZE-ESI-μTOF MS method for nitrated allergens requires an individual investigation of key parameters both in CZE separation and ESI as well as the evaluation of their effect on resolution (*R*_S_), spray stability, and MS signal intensity of nitrated variants. Some preoptimized parameters, such as position of spray needle relative to the sprayer body, protrusion of the CE capillary from the spray needle, temperature, and flow rate of DG, and spray voltage were maintained throughout the optimization. During the entire optimization the same solution of nBet v 1a was analyzed that was derived from a 1:1 ratio (mol/mol) PN to Tyr in the reaction mixture with a final nominal protein concentration of 0.4 mg/mL. This ensures comparability of individual optimization steps.

### 3.1 BGE optimization

The maintenance of high resolution and separation efficiency constitutes an essential challenge when transferring CZE-UV to CZE-ESI-MS. This is related to a restricted selection of volatile BGEs and additives in MS [42, 44]. Moreover, an increase in ionic strength as frequently applied in CZE-UV to reduce protein adhesion and improve efficiency results in ion suppression in MS [44, 53]. Therefore, an applicable balance between these counteracting effects has to be considered in MS.

#### 3.1.1 Optimization of pH of volatile BGE within pH 6–9

Previous optimization strategies for the separation of Bet v 1a and modified variants covering a p*I* domain similar to nBet v 1a have revealed a pH regime around 6.5 most advantageous [28, 30]. This is related to the experimentally determined p*I* of ∼5.0 for Bet v 1a [29]. Stepwise nitration entails no charge losses, but only changes in the pK_a_ [6]. Thus, selection of the pH optimum is crucial since it determines *R*_S_, selectivity, and peak shape [54]. Based on previous results [28], a pH domain between 6 and 9 was pretested for nBet v 1a. Hence, NH_4_HCO_3_ was selected due to its pK_a_ values and the improved performance in comparison with other volatile BGEs, i.e. ammonium acetate and -formate (data not shown). Based on the facile degassing at pH 6.5, the related pH drift [Bibr b32] and the screening results (data not shown), an initial pH of 8.0 was selected for subsequent optimization of NG and SL flow rates.

### 3.2 Flow rate of SL

**Figure 1:**
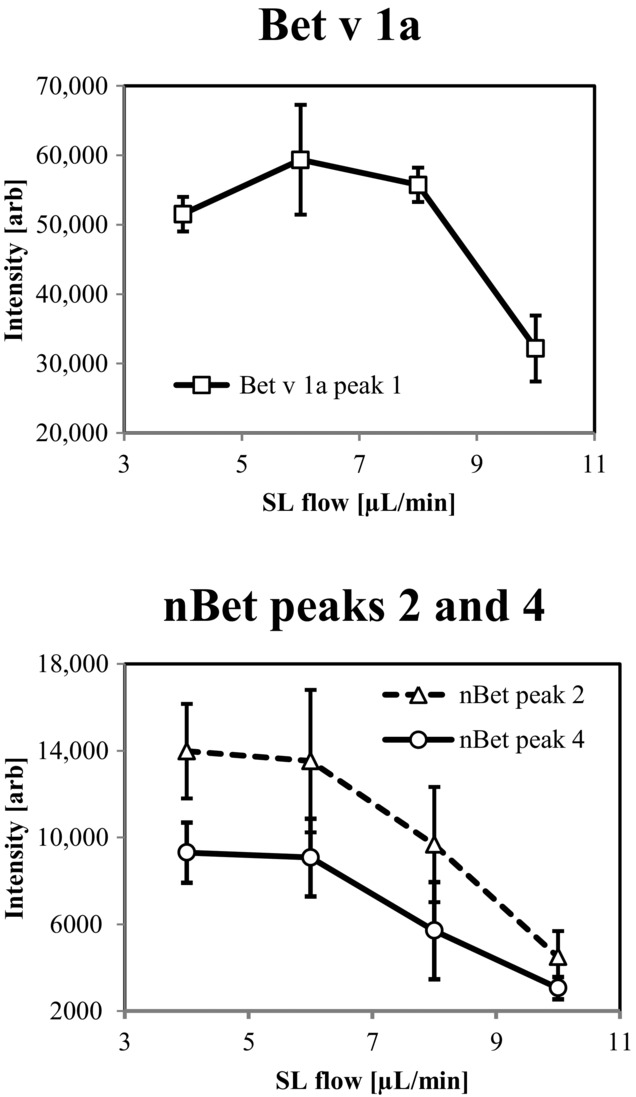
Optimization of SL flow and its influence on the intensity of nonmodified Bet v 1a, and single nitrated Bet v 1a variants (peak 2 and 4). For peak annotations see Fig. 3. Sample: 0.4 mg/mL Bet v 1a nitrated with 1:1 n/n PN to Tyr. CZE separation: BGE: 25 mmol/L NH_4_HCO_3_, pH 8.04. 25 kV, 25°C. Capillary: bare fused silica 100.5 cm. Injection: 35 mbar, 10 s. ESI: SL: methanol/H_2_O/FA 75%:24.9%:0.1% v/v/v. NG flow 0.4 bar. DG: 4 L/min, 190°C. MS-transfer capillary voltage: –4.5 kV (positive ion mode). Error bars represent ± SD (*n* = 3).

The SL flow rate was varied from 2 to 10 μL/min with SL composition specified in Fig. 1. Within this flow range mainly the baseline stability (measured as total ion current) and peak intensities were affected. A flow rate of 2 μL/min showed pronounced fluctuations of the total ion current, affected signals, and was unable to yield a stable spray. Therefore, only flow rates ≥4 μL/min were considered. Figure 1 demonstrates the influence of SL flow on signal intensities of the most abundant peaks, i.e. nonmodified Bet v 1a and the most prominent nBet v 1a variants (see Section 3.4, peaks 2 and 4 in Fig. 3C). For nonmodified Bet v 1a (Fig. 1) only minor intensity differences were encountered between 4 and 8 μL/min. Signal intensities for the most prominent nBet v 1a species (see peaks 2 and 4, Fig. 3) were considerably lower. For selected nBet v1a variants, signal intensities at 4 and 6 μL/min were equivalent, but due to improved baseline stability, 6 μL/min was selected for further optimization. At 8 and 10 μL/min signal intensities for nBet v 1a decreased (Fig. 1).

**Figure 2:**
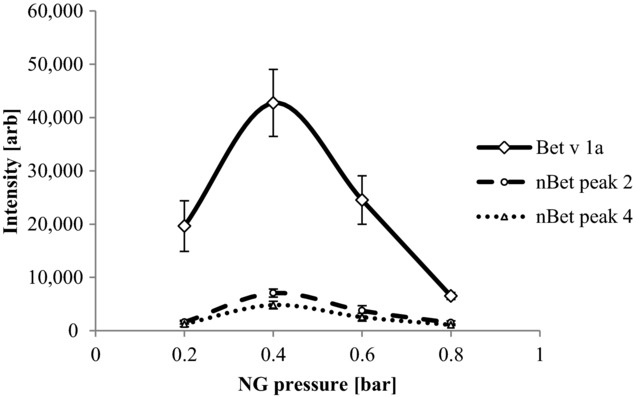
Influence of NG on the intensity of target allergens, i.e. nonmodified Bet v 1a (peak 1) and prominent nBet v 1a variants (peak 2 and 4). For peak annotations see Fig. 3. SL flow was 6.0 μL/min. For all other settings see Fig. 1.

**Figure 3:**
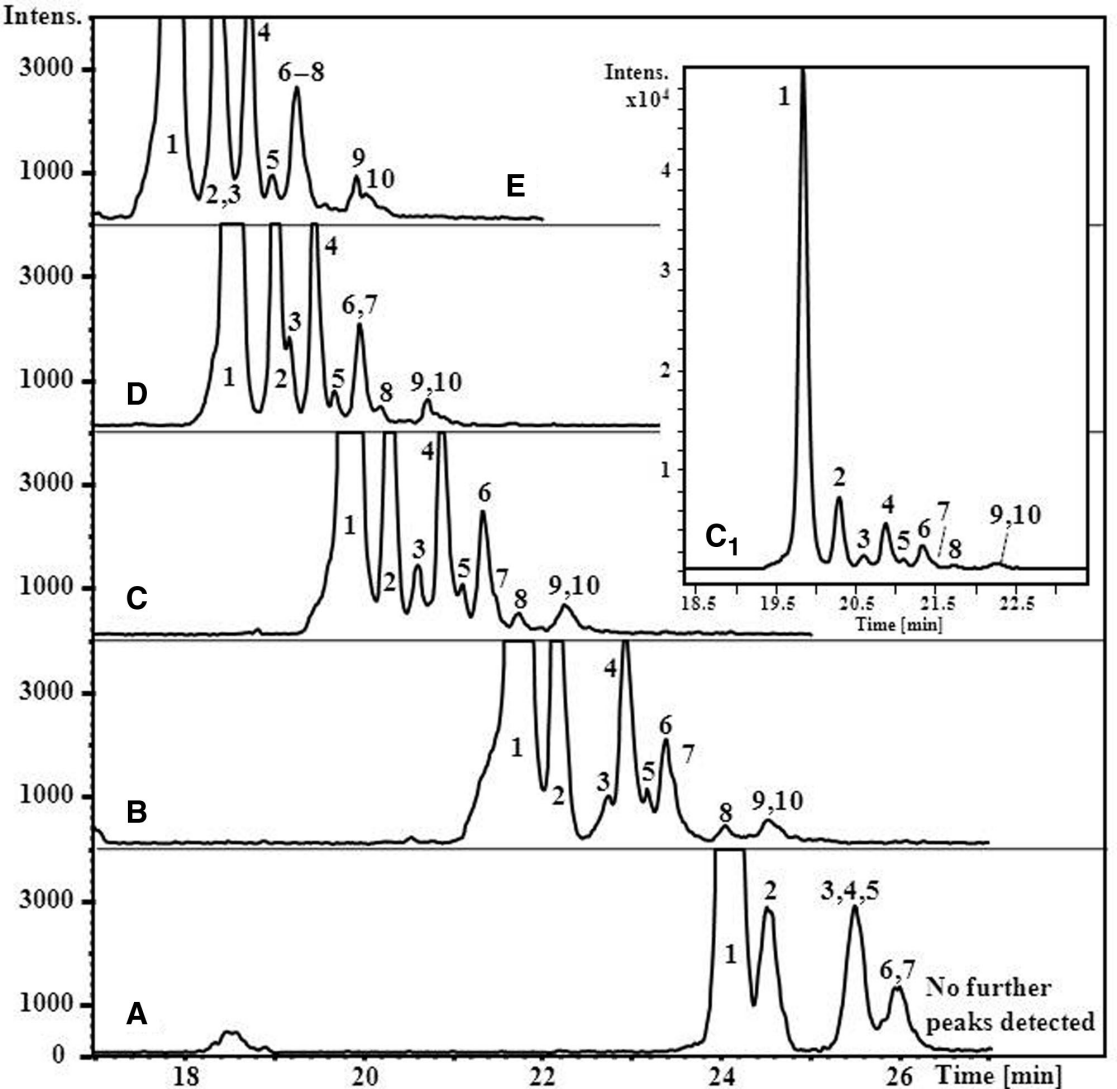
BGE optimization for CZE separation. Overlay of base peak electropherograms (BPEs) of the 1:1 nitrated Bet v 1a sample (with 0.4 mg/mL) measured by CZE-ESI-TOF MS testing different pH values for 25 mmol/L NH_4_HCO_3_: (A) 7.08, (B) 7.28, (C) 7.48, (D) 7.68, and (E) 7.88. SL flow was 6.0 μL/min; NG: 0.4 bar. For all other settings see Fig. 1. Peak annotations: 1: nonmodified Bet v 1a; 2–4: single nitrated nBet v 1a variants; all other peaks are identified in Table 2. For trace C, the entire BPE is given. Peaks 8–10 were not detected in trace A even at extended analysis time.

### 3.3 Nebulizer gas optimization

The ratio between flow velocity of the BGE and the SL- and NG flow, respectively, provides the main contribution to the suction effect, which depends on geometry of the CZE-ESI interface [42]. Hence, an appropriate optimization of NG flow is obligatory since it influences *R*_S_, separation efficiency, ionization, spray stability, and thus peak intensity. In order to exclude fluctuations of *t*_m_ by pH drifts the BGE was replenished every run. Figure 2 shows the NG pressure optimization between 0.2 and 0.8 bar and its effect on signal intensities for Bet v 1a and two prominent nBet v 1a variants. Moreover, the increase in the NG flow caused a linear decrease of the *t*_m_ of analytes over the four tested pressure values due to the suction effect. For instance, Bet v 1a was accelerated from 1116.0 s (0.2 bar) to 932.3 s (0.8 bar) accompanied by loss in *R*_S_. Equivalent effects were observed for nBet v 1a variants (data not shown). In combination with spray parameters and selected SL flow (see Section 3.2), NG provided highest peak intensities at 0.4 bar (Fig. 2).

### 3.4 Optimization of volatile BGE

#### 3.4.1 Refined pH optimization

Based on the results of Section 3.1.1, the range between pH 7.1 and 7.9 was further investigated in increments of ∼0.2 pH units (Fig. 3). As evident from trace A (Fig. 3) only four fractions of nBet v 1a were resolved at pH 7.08 even at increased *t*_m_. The apparent comigration of several fractions is most likely due to adsorptive effects or unfavorable differences in effective electrophoretic mobilities. A stepwise pH increase considerably improved *R*_S_ (trace B) and finally allowed for the distinction of nine fractions, whereby fractions 9 and 10 were not resolved (trace C). A further increase to pH 7.68 and 7.88 entailed a loss in *R*_S_ or even a merging of several nitrated variants, respectively. In general, the pH increase merged peaks 2 and 3 and additionally impaired their *R*_S_ from peak 4. Similarly, peaks 6–8 merged. On the contrary, *R*_S_ of peaks 4 and 5 and of peaks 1 and 2 improved and one minor subfraction of peak 9 occurred (Fig. 3, traces D, E). Based on these results, pH 7.48 provided the best selectivity for nBet v 1a within the tested pH range, although fraction 10 was not entirely resolved. Peak identities are discussed in detail in Section 3.6 for the elaborated CZE-ESI-TOF MS method. As evident, minor pH changes will affect selectivity and thus an accurate pH adjustment and maintenance are essential to achieve *R*_S_ given in Fig. 3, trace C.

#### 3.4.2 Optimization of BGE concentration

A further step in BGE optimization encompassed the concentration while maintaining pH 7.48. The NH_4_HCO_3_ concentration was varied between 10 and 50 mmol/L. This increase in the concentration also increased *t*_m_ due to a reduction of the EOF. Simultaneously, the signal intensity in MS was reduced to 51.7–54.5% (for 25 mmol/L) and further to 17.7–24.2% (for 50 mmol/L; *n* = 3 in either case) of the values for 10 mmol/L considering the most prominent peaks, i.e. nonmodified Bet v 1a (peak 1) and two single nitrated variants (Fig. 3C, peaks 2 and 4). This is most likely due to ion suppression (data not shown). Consistent with these results, 50 mmol/L NH_4_HCO_3_ was excluded. Instead, 10 mmol/L NH_4_HCO_3_ was chosen for further optimization due to higher MS signal intensities in comparison with 25 mmol/L. An extension to lower BGE concentrations was not approved due to the encountered repeatability problems of *t*_m_ (data not shown).

### 3.5 SL optimization

The composition of the SL, which generally implements an organic solvent (preferably methanol or IP) and mostly a volatile acid, directly influences the spray process, but also the protonation degree and hence the signal intensity of intact proteins. Additionally, organic modifiers can affect the protein conformation resulting in an increased exposure of possible protonation sites and thus lower *m/z* states [55, 56]. Proper optimization of SL is obligatory to establish stable and sensitive CZE-ESI-MS hyphenation.

#### 3.5.1 Content of organic solvent in SL

Variations in SL composition and related solvent properties, e.g. pH, polarity, or surface tension [57], were shown to induce changes in the protein conformation and thus also in charge state distribution (CSD) in ESI-MS [44, 55, 56]. The current work addresses the influence of methanol and IP by testing different volume ratios, respectively. Formic acid that was added for protein protonation in positive ion mode was initially kept at 0.1% v/v.

##### 3.5.1.1 Methanol

**Figure 4:**
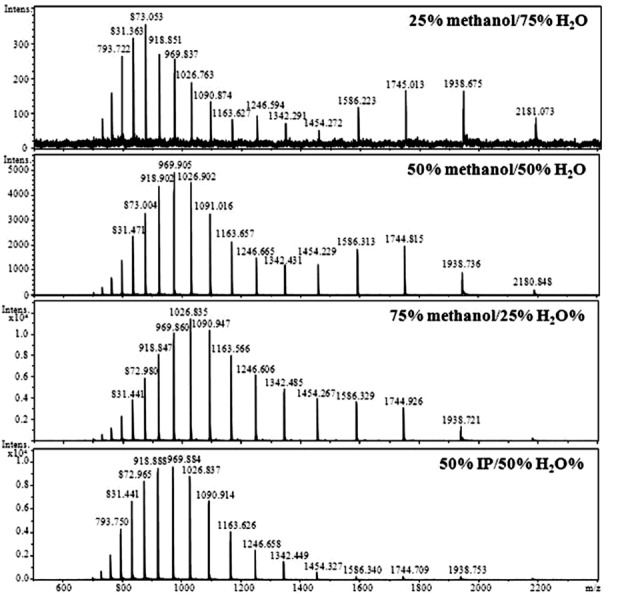
Effect of methanol and IP content in SL on CSD of Bet v 1a. BGE: 10 mmol/L NH_4_HCO_3_, pH 7.50; SL composition: 0.1% v/v FA in ultrapure water with (A) 25%, (B) 50%, (C) 75% v/v methanol, and (D) 50% v/v IP. All other settings were according to Fig. 3.

For 25% v/v methanol, a bimodal CSD was observed for nonmodified Bet v 1a (Fig. 4). Dobo and Kaltashov [58] have revealed conformation-specific CSDs assigned as “basis distributions,” with bi- and multimodal CSDs referring to overlapping “basis distributions” of coexisting conformations. In our case, increased methanol content, i.e. 50% and 75% v/v, progressively shifted the CSD toward unimodal shape (Fig. 4). The same effect was observed for nBet v 1a variants (data not shown). This is in line with previous discussions showing that methanol can relax protein conformations, increase the accessibility of protonation sites, and consistently shift CSDs toward lower *m/z* [55]. In addition, a higher methanol content, i.e. 75% v/v, increased also signal intensities (Table 1). At contrary, 100% v/v methanol dramatically reduced MS signal intensities and increased the baseline (data not shown).

**Table 1:** Influence of SL composition on signal intensity in CZE-ESI-TOF MS (*n* = 3) for Bet v 1a nitrated with 1:1 PN to Tyr with a final concentration of 0.4 mg/mL

Compound	Ratio v/v	Signal intensity [arb]		
		
		Nonmodified Bet v 1a	1× Nitrated Bet #1	1× Nitrated Bet #3
		Mean ± SD	Mean ± SD	Mean ± SD
*SL composition: 0.1% v/v FA and ultrapure water with*				
Methanol	50%	7503 ± 285	457 ± 75	274 ± 77
	75%a)	9562 ± 1687	1247 ± 218	848 ± 101
Isopropanol	50%	8194 ± 1629	1642 ± 759	1087 ± 438
	75%	5922 ± 142	1457 ± 87	956 ± 65
*SL composition: 75% v/v methanol in ultrapure water with*				
Formic acid	0.05%	15,499 ± 475	2192 ± 58	1428 ± 94
	0.1%a)	14,316 ± 95	2306 ± 132	1538 ± 151
	0.5%	7381 ± 303	1514 ± 77	969 ± 18
	1.0%	3786 ± 356	884 ± 52	529 ± 56

a) Although SL composition and sample were identical, slightly different intensities were encountered. This is related to a measurement at different days. The optimization of organic solvents and of formic acid was done at separate days, respectively.

##### 3.5.1.2 Isopropanol

Beside methanol [59], IP has been frequently applied in SL [33, 40, 60]. Therefore, the optimization strategy was replicated with IP, again screening concentrations of 25, 50, 75, and 100% v/v. Contrary to methanol, the CSD with IP was shifted toward lower *m/z* with symmetric distribution irrespective of the selected IP content (Fig. 4). The effects outlined for the CSD of Bet v 1a were observed equivalently for prominent nBet v 1a variants and thus not depicted separately. As for methanol, 25% and 100% v/v IP provided poor signal intensities. At 50% and 75% v/v, IP provided nearly equivalent intensities for both prominent nBet v1a variants (peaks 2 and 4, Fig. 3C; Table 1). For nonmodified Bet v 1a, the signal intensity was reduced by ∼28% when switching from 50 to 75% v/v IP. In comparison with 50% v/v methanol, signal intensities for nBet variants were 3.6–4.0-fold higher for 50% v/v IP at the expense of increased standard deviation of signals for peak 2 (Table 1). Nonmodified Bet v 1a provided similar signals, but again a higher standard deviation for IP. At 75% v/v, signal intensities of nBet v 1a variants were similar for methanol and IP, and only slightly lower than those for 50% v/v IP. In case of nonmodified Bet v 1a, 75% v/v methanol provided a 1.6-fold higher signal than 75% v/v IP (Table 1).

In general, an increase from 50 to 75% v/v IP reduced the signals for nBet v 1a marginally, but remarkably for nonmodified Bet v 1a. On the contrary, increasing methanol from 50 to 75% v/v increased all signal intensities. Despite the fact, that 50% v/v IP shows higher signals, 75% v/v methanol was considered as an optimum due to reduced standard deviations for nitrated allergens. Thus, optimization of FA was done for 75:25% v/v methanol to water (Table 1). The differences in CSD as well as signal intensities in response to increasing content of methanol or IP in SL are probably related to the different polarity of both tested solvents.

#### 3.5.2 Formic acid content in SL

To assure appropriate ionization of target allergens in positive ion mode, a volatile acid has to be added to the SL. The concentration should not exceed a certain limit, to avoid instability of electrospray and ion suppression [59, 61]. FA was varied between 0.05 and 1.0% v/v in a SL composed of 75% v/v methanol and 25% v/v ultrapure water. Table 1 shows highest signal intensities of nBet v 1a variants at 0.1% v/v FA. Although 0.05% v/v FA showed a slightly higher intensity (by 8.3%) for nonmodified Bet v 1a, 0.1% v/v FA was selected since nBet v 1a represent the target analytes. At 0.5 and 1.0% v/v FA signals were considerably reduced for all analytes (Table 1).

### 3.6 Characterization of nitrated allergens

**Figure 5:**
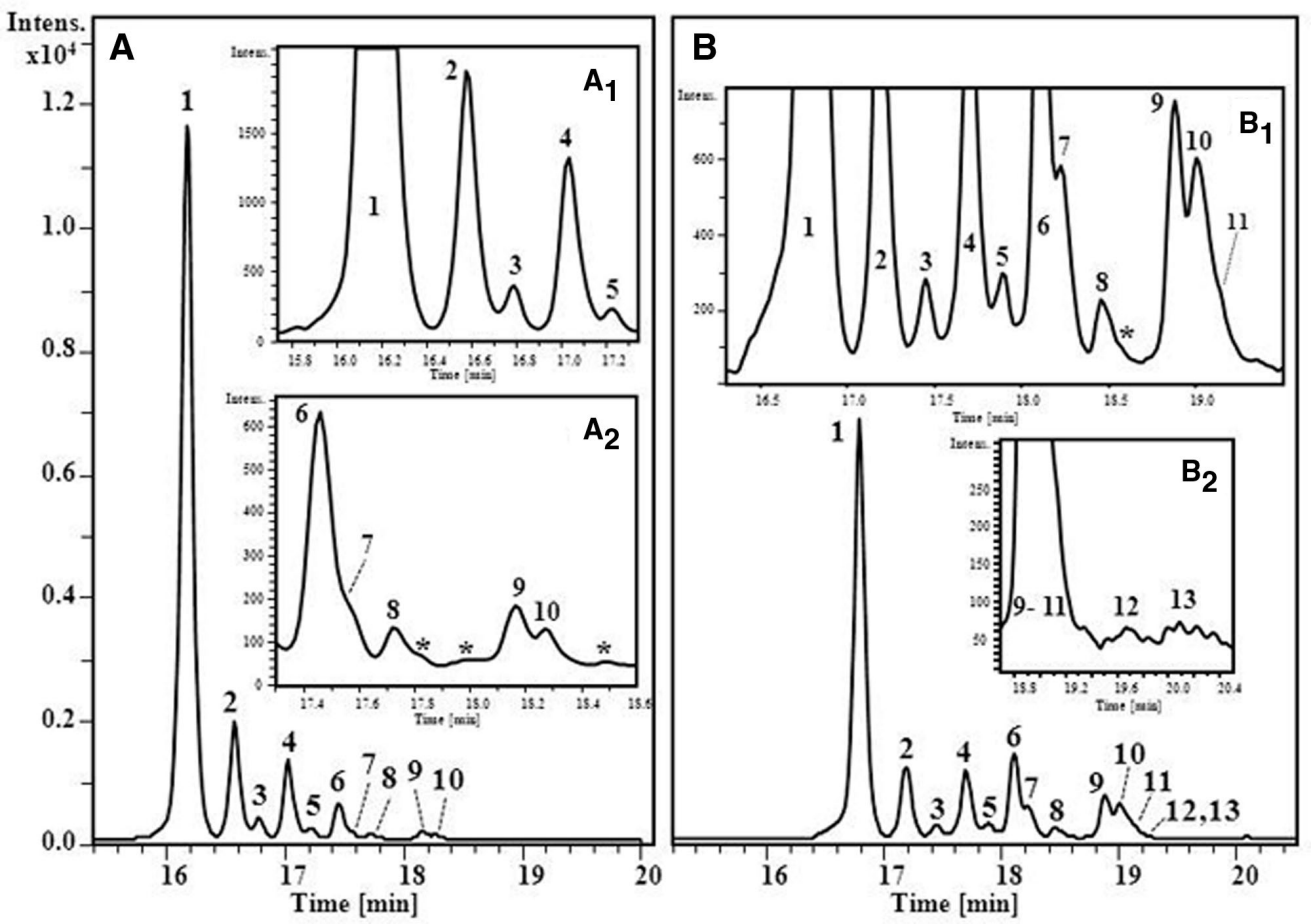
Comparison of differently nitrated Bet v 1a samples. The applied ratio (mol/mol) between PN and Tyr in the respective nitration mixture was (A) 1:1, and (B) 5:1. Traces give BPEs of the respective sample. Inserts (A_1_–B_2_) provide details. BGE: 10 mmol/L NH_4_HCO_3_, pH 7.50; SL: 0.1% v/v FA/75% v/v methanol in ultrapure water with 6.0 μL/min; NG: 0.4 bar. All other settings were according to Fig. 4. For peak annotations (1–13) see Table 2. *indicates low intensity signals where deconvolution failed to provide masses.

In addition to the nitrated sample derived from a 1:1 (mol/mol) ratio of PN to Tyr in the reaction mixture, another sample with 5:1 ratio was characterized by CZE-ESI-TOF MS. Results for both samples are depicted in Fig. 5 and surveyed in Table 2. In either case, CZE-ESI-TOF MS revealed no oxidation of the only thiol-containing residue, i.e. Met 139 [http://www.uniprot.org/uniprot/P15494], under the selected nitration conditions but merely M_r_ increments of 44.99 that refer to nitration (Fig. 5, Table 2). Thus, the CO_2_ content in the reaction mixture (delivered by bicarbonate) is sufficient to prevent oxidation and foster preferential nitration at Tyr [17, 62].

**Table 2:** Comparison of nitrated recombinant Bet v 1a samples derived from reaction mixtures containing different ratios between PN and Tyr, respectively

Peak Nra)	Allergen variantb)	Theor. mass M_r_	1:1 Nitration samplec)			5:1 Nitration sample^d)^		
				
			*t*_m_[s]	Exp. mass/error	Relative	*t*_m_[s]	Exp. mass/error	Relative
				M_r_ [ppm]	intensitye)		M_r_ [ppm]	intensity
1	Bet v 1a	17439.600	970.7	17439.933/19.1	100	1007.3	17439.891/16.7	100
2	1× nitro #1	17484.598	995.4	17484.910/17.8	17	1031.1	17484.821/12.8	18
3	1× nitro #2	17484.598	1007.3	17484.878/16.0	4	1046.6	17485014/23.8	4
4	1× nitro #3	17484.598	1022.0	17484.937/19.4	11	1061.3	17484.923/18.6	17
5	2× nitro #1	17529.600	1033.9	17529.927/18.9	2	1073.2	17529.769/9.6	4
6	2× nitro #2	17529.600	1047.6	17529.939/19.6	5	1086.0	17529.987/22.1	20
7	3× nitro #1	17574.598	1054.9	17575.111/29.4	2	1093.3	17574.953/20.2	8
8	2× nitro #3	17529.600	1064.1	17529.959/20.7	1	1107.1	17529.889/16.5	3
9	3× nitro #2	17574.598	1089.7	17575.275/38.7	2	1132.7	17574.994/22.5	11
10	4× nitro #1	17619.596	1097.0	17620.928/75.8	1	1140.0	17620.076/27.2	9
11	5× nitro #1	17664.594	n.d.f)	n.d.	n.d.	1148.2	17664.886/16.5	4
12	5× nitro #2	17664.594	n.d.	n.d.	n.d.	1180.3	17665.300/40.0	1
13	6× nitro #1	17709.592	n.d.	n.d.	n.d.	1205.0	17710.600/56.9	1

a) Peak numbers refer to annotations given in Fig. 5.

b) Variants #1–3 refer to nBet v1a variants of nominally identical mass, but different *t*_m_ (see Fig. 5).

c) and d) Refer to nitrated Bet v 1a samples derived from reaction mixtures containing PN:Tyr ratios of 1:1 and 5:1, respectively.

e) Refers to Bet v 1a of the respective sample that is assigned 100%.

f) Not determined since the S/N in BPE was too low to allow for unambiguous identification.

Both samples provided protein peaks of corresponding masses after deconvolution that prove incremental nitration. In either case, the nonmodified source Bet v 1a was not nitrated entirely and still present (peak 1, Fig. 5). Both samples possessed three peaks with nominally equivalent masses (17484.821–17485.014), indicating the theoretical ΔM_r_ of 44.99 from the nonmodified isoform (Table 2) expected for single nitrated variants. Their *t*_m_ differences are most likely caused by site-specific Tyr nitration inducing slightly variable p*I* and hydrodynamic radii. Site-specific nitration propensity explains also the differences in abundance (excluding variant-specific differences in ionization efficiency) due to different accessibilities of Tyr residues. Most likely, Tyr residues nitrated in peaks 2 and 4 (Fig. 5) possess a similar accessibility, but differ in their final impact on p*I* and/or hydrodynamic radius. Consistently, the nitration propensity for the Tyr position addressed in peak 3 is lower. Similar effects are also observed for double nitrated variants, whereby assumed combinations of two high and one low propensity Tyr sites, respectively, should theoretically result in one prominent and two less abundant peaks (Fig. 5, peaks 5, 6, 8; Table 2). Additionally, three- and fourfold nitrated variants are identified in the 1:1 sample. The sample derived from the 5:1 reaction mixture includes three further variants, i.e. two five- and one sixfold nitrated species. The low abundances of these variants (peaks 11–13, Fig. 5B, B_2_ and Table 2) indicate the restricted accessibility of related Tyr residues. In summary, the sample derived from the 5:1 reaction mixture contains 12 different nBet v 1a variants. With the exception of the single nitrated species, nitrated variants possess two- to ninefold higher relative abundances in the 5:1 sample when compared to the 1:1 sample (Table 2). Thereby, three- and fourfold nitrated variants (peaks 9 and 10) are over proportionally increased in the higher nitrated sample (Fig. 5A, B and Table 2). Generally, results indicate a tendency toward increased nitration grade with higher PN concentrations. The slight changes in *t*_m_ but also minor changes in *R*_S_ are related to the different sample composition, but also to slight changes in the volatile BGE due to the delicacy of the pH (see Fig. 3). Mass accuracies were between 9.6 and 27.2 ppm. Higher errors are related to the low abundance of minor constituents, primarily those of higher nitration grades (Table 2).

## 4 Concluding remarks

A CZE-ESI-TOF MS method has been developed and optimized addressing nitrated variants of the major birch pollen allergen Bet v 1a created by reaction with PN. The BGE optimization revealed 10 mmol/L ammonium bicarbonate with pH 7.50 essential to resolve and detect minor nitration variants. Deviations by 0.2 pH units from this optimum impaired *R*_S_ substantially. An appropriate composition of the SL, comprising type and content of organic solvent and the volatile acid, in concert with adjusted flow rates of SL and NG was crucial for maintaining *R*_S_, establishing a stable electrospray and appropriate ionization conditions, while simultaneously preventing detrimental contributions of ion suppression and combined suction/dilution effects. Two aliquots of a recombinant Bet v 1a batch were nitrated by different PN-to-Tyr ratios in the reaction mixture and compared by the elaborated CZE-ESI-TOF MS method. For the 5:1 sample, results demonstrated a shift toward species with higher nitration level, i.e. three- to sixfold nitrated variants, while relative abundances of single and double nitrated variants were unaffected. The explanation of nitration profiles requires both understanding of the nitration mechanism and the protein structure. Evidently, the key for profile interpretation is conformational accessibility of Tyr residues that governs site-specific nitration. Profiles demonstrated higher *t*_m_ with increased nitration levels most likely due to a reduction of p*I* in the train of incremental nitration. Within coexisting species of identical nitration grade, site-specific influences on the p*I* and possibly on the domain conformation presumably entail mobility differences and allow for a distinction. The elaborated CZE-ESI-TOF MS method represents a selective tool for the characterization of nitrated allergen variants that occupy a narrow p*I* range. This will enable an essential progress in the characterization, and mid-term standardization, of such products that are required by immunologists to reveal and confirm their postulated higher allergenicity in comparison with nonmodified counterparts.

## Abbreviations

Bet v 1a: major birch pollen allergen from *Betula verrucosa*, isoform 1a
BPE: base peak electropherogram
CSD: charge state distribution
DG: dry gas
FA: formic acid
IP: isopropanol
nBet v 1a: nitrated Bet v 1a variant
NG: nebulizer gas
PN: peroxynitrite
*R*_S_: resolution
SL: sheath liquid
*t*_m_: migration time
Tyr•: tyrosine radical
